# Mass drug administration can be a valuable addition to the malaria elimination toolbox

**DOI:** 10.1186/s12936-019-2906-8

**Published:** 2019-08-22

**Authors:** Thomas P. Eisele

**Affiliations:** Department of Tropical Medicine, Center for Applied Malaria Research and Evaluation, Tulane School of Public Health and Tropical Medicine, New Orleans, LA USA

**Keywords:** Malaria, Elimination, Mass drug administration (MDA)

## Abstract

The Global Technical Strategy 2016–2030 of the World Health Organization (WHO) has the ambitious goal of malaria being eliminated from at least 35 countries by 2030. However, in areas with once-stable malaria transmission, the reservoir of human infection may be intermittently symptomatic or fully silent yet still lead to transmission, posing a serious challenge to elimination. Mass drug administration (MDA), defined as the provision of a therapeutic dose of an effective anti-malarial drug to the entire target population, irrespective of infection status or symptoms, is one strategy to combat the silent human reservoir of infection. MDA is currently recommended by the WHO as a potential strategy for the elimination of *Plasmodium falciparum* malaria in areas approaching interruption of transmission, given the prerequisites of good access to case management, effective vector control and surveillance, and limited potential for reintroduction. Recent community randomized controlled trials of MDA with dihydroartemisinin–piperaquine, implemented as part of a comprehensive package of interventions, have shown this strategy to be safe and effective in significantly lowering the malaria burden in pre-elimination settings. Here it is argued that effectively implemented MDA should be kept in the elimination toolbox as a potential strategy for *P. falciparum* elimination in a variety of settings, including islands, appropriate low transmission settings, and in epidemics and complex emergencies. Effectively implemented MDA using an ACT has been shown to be safe, unrelated to the emergence of drug resistance, and may play an important role in sufficiently lowering the malaria burden to allow malaria transmission foci to be more easily identified, and to allow elimination programmes to more feasibly implement case-based surveillance and follow-up activities. To be most impactful and guard against drug resistance, MDA should use an ACT, achieve high programmatic coverage and adherence, be implemented when transmission is lowest in areas of limited risk of immediate parasite reintroduction, and must always be implemented only once good access to case management, high coverage of effective vector control, and strong surveillance have been achieved. If these considerations are taken into account, MDA should prove to be a valuable tool for the malaria elimination toolbox.

## Background and evidence for mass drug administration

Despite malaria remaining a major cause of morbidity and mortality throughout much of the world [1], many endemic countries have had tremendous success against the disease over the past decade and are now targeting malaria elimination. The World Health Organization (WHO) Global Technical Strategy 2016–2030 has the ambitious goal of malaria being eliminated from at least 35 countries by 2030 [2]. The human parasite reservoir is an important factor that fuels ongoing transmission and poses a serious challenge for elimination. In areas that once had stable malaria transmission, this reservoir of human infection may be intermittently symptomatic or fully silent yet still contribute to transmission [3–7]. A strategy to combat this parasite reservoir is mass drug administration (MDA), defined as the provision of a therapeutic dose of an effective anti-malarial drug to the entire target population, irrespective of infection status or symptoms. MDA is recommended by the WHO as a potential strategy for the elimination of *Plasmodium falciparum* malaria in areas approaching interruption of transmission, as well as the Greater Mekong Subregion (GMS) where multidrug resistance is present, given the prerequisites of good access to case management, effective vector control and surveillance, and limited potential for reintroduction [8]. Its time-limited use has also been recommended by the WHO during malaria epidemics and complex emergencies in malaria endemic regions. In areas of moderate to high transmission, the WHO acknowledges MDA may yield a short-term reduction in malaria burden, but more evidence is required for it to be a recommended strategy in such settings.

For some time, MDA has been a recommended part of an integrated strategy for eliminating many neglected tropical diseases, including lymphatic filariasis [9], onchocerciasis [10] and schistosomiasis [11]. The effectiveness of MDA against malaria has been assessed in studies dating back to the 1950s [12–18]. Although there is limited evidence to date from rigorous controlled trials that MDA can successfully interrupt malaria transmission [19], there are several less rigorous observational studies that have shown MDA, in combination with vector control and surveillance improvements, to have interrupted transmission for up to 6 months [20]. MDA has also interrupted malaria transmission for sustained periods among isolated island populations [21, 22].

More recent community randomized controlled trials of MDA with the long-acting anti-malarial dihydroartemisinin–piperaquine (DHAP), along with a package of interventions, have shown this strategy to be safe and effective at significantly lowering malaria prevalence and incidence in pre-elimination settings [23–25]. A recent trial in Zambia in a low transmission area [prevalence in children under 5 years of age < 10%] evaluated 2 rounds of MDA-DHAP on top of a standard of care of improved access to case management, vector control and surveillance [23]. *Plasmodium falciparum* parasite prevalence in children under 5 years of age in the MDA arm significantly decreased from 7.7% at baseline during peak transmission, to < 1% during the next year’s peak season 3-months post-MDA, representing an 87% larger decrease compared to the standard of care. These findings were used by the Zambia National Malaria Elimination Centre to add MDA-DHAP to its package of interventions in areas targeting subnational elimination [26].

In the recent cross-over trial in four countries in the GMS, three rounds of MDA-DHAP on top of a standard of care of improved access to case management and surveillance significantly reduced *P. falciparum* prevalence from 5.1% at baseline to < 1% at follow-up, representing a significantly greater decline compared to the standard of care [24]. Additional analysis of the Myanmar data has suggested a potential community effect of MDA, whereby individuals in communities that received high coverage of MDA, yet did not receive the drugs themselves, still benefited from its effect [27].

Mathematical models support these more recent findings and suggest that when high effective coverage is achieved in settings with *P. falciparum* prevalence at or below 5% and there is limited risk of reintroduction, MDA can result in a substantial reduction in prevalence and incidence, which, when combined with high coverage of effective vector control and good access to case management, can be sustained for substantial periods of time after cessation of MDA [28, 29].

## The argument for keeping MDA in the malaria elimination toolbox

Why has MDA for malaria been considered an historical failure? Often, these older MDA efforts were very time-limited, achieved poor coverage and adherence, and were not delivered as a package of interventions including good vector control and access to quality case management. Poor adherence and monotherapy may have contributed to drug resistance [18, 30–32]. In addition, the lack of empirical evidence that MDA can achieve sustained interruption of transmission has contributed to reluctance among many in the malaria community to support the use of MDA [33]. Contrary to this skepticism, newer evidence argues for effectively implemented MDA to be kept in the *P. falciparum* elimination toolbox for use in a variety of settings, including islands, appropriate low transmission settings, and in epidemics and complex emergencies.

This argument is based on several lines of evidence. First, the use of an artemisinin-based combination treatment (ACT), such as DHAP for MDA for *P. falciparum* has been shown to be safe [19, 20, 22, 23, 34, 35]. Although piperaquine has the potential for a dose-dependent prolongation of the QT interval when monitored by electrocardiogram, a recent systematic review found that among 757,000 DHAP treatment regimens administered, there was no excess risk of sudden cardiac death compared to those that did not receive the drug [34].

Second, while poorly implemented MDA using a monotherapy that results in subtherapeutic dosing may contribute to drug resistance [30, 31], there is no evidence that effectively implemented MDA with an ACT has led to drug resistance [18, 20]. Two recent publications suggest that MDA using an ACT (containing artemisinin which has a very short half-life, along with a longer half-life partner drug) administered all at once in a low transmission setting with high coverage and adherence, is highly unlikely to lead to the emergence of de novo drug resistance [36, 37].

Third, the two recent MDA-DHAP trials from Zambia and the GMS showed a reduction in *P. falciparum* prevalence from a baseline of 5–10% down to < 1%, along with concomitant reduction in malaria incidence [23, 24]. Importantly, the results from Zambia show the reduction in prevalence to < 1% to have been sustained for up to 15-months after the last MDA campaign round as a result of continued high coverage of vector control, good access to case management and improved surveillance (unpublished data). Although malaria transmission was not interrupted in either trial, achieving and sustaining an infection prevalence below 1% is important for malaria elimination programmes. The very low level of transmission that can be achieved by MDA in such settings likely allows transmission hotspots to be more easily identified and targeted due to increased spatial heterogeneity. [38–40]. The lower transmission achieved also allows an elimination programme to more feasibly perform case investigations and follow-up activities, enabling the establishment of a case-based surveillance system, a prerequisite for malaria elimination [41]. MDA is also a quintessential example of an intervention that requires strong community engagement and participation, which when achieved can strengthen the overall malaria elimination programme. The actual implementation of the MDA campaign rounds also helps build significant capacity in an elimination programme to undertake large-scale elimination activities.

Based on systematic historical reviews, results from more recent trials, and modelling, there is growing consensus on the “who, what, when and where” to consider when deciding to implement an MDA strategy. There is consensus, as reflected in WHO policy recommendations, that MDA should be time limited and only be implemented once high coverage of effective vector control, good access to case management, and a strong surveillance system are in place and maintained during and after MDA [8, 19, 20, 42]. Not only will these core interventions maximize the impact of MDA on burden reduction, they are also central to maintaining the gains once MDA is stopped [29]. MDA should be limited to areas of low transmission, preferably under 5-10% prevalence, to maximize its impact and mitigate drug resistance [29, 36, 37]. To further protect against drug resistance and maximize impact, MDA should use an ACT administered by directly-observed therapy to maximize adherence, with rounds conducted over a short period of time using door-to-door campaigns during or at the end of the dry season [18, 20, 29, 36, 37]. The optimal number of MDA rounds remains unclear, other than using as many as needed to achieve high (> 80%) population coverage [20, 28, 29]. Furthermore, there is strong consensus that extensive community and stakeholder engagement is required to achieve high MDA coverage, optimally based on high-quality formative research [20, 24, 33, 43]. Attention should also be paid to highly mobile populations that may be missed over multiple campaign rounds, and who may also be a source of reintroduction of parasites back into the community [24]. MDA should be limited to areas with limited potential for quick parasite reintroduction, and where possible should target sources of malaria exportation to neighbouring areas [29, 35, 40, 44]. If these considerations are taken into account, MDA should prove to be a valuable tool for the malaria elimination toolbox.

## Conclusion

We argue that effectively implemented MDA should be kept in the malaria elimination toolbox for use in a variety of settings, including islands, appropriate low transmission settings, and in epidemics and complex emergencies. In areas of low transmission when combined with high coverage of vector control, good access to case management and strong surveillance, MDA with an ACT like DHAP has been shown to be safe and effective at significantly reducing the *P. falciparum* malaria burden, allowing elimination programmes to more feasibly perform individual case-based surveillance and focus limited resources on targeting remaining transmission hotspots.

## Data Availability

Not applicable.

## Abbreviations

ACT: artemisinin-based combination treatment
DHAP: dihydroartemisinin–piperaquine
GMS: Greater Mekong Subregion
MDA: mass drug administration
WHO: World Health Organization

## References

[1] WHO (2018). World Malaria Report 2018.

[2] WHO (2015). Global Technical Strategy for Malaria 2016–2030.

[3] Nguyen TN, von Seidlein L, Nguyen TV, Truong PN, Hung SD, Pham HT (2018). The persistence and oscillations of submicroscopic *Plasmodium falciparum* and *Plasmodium vivax* infections over time in Vietnam: an open cohort study. Lancet Infect Dis..

[4] Okell LC, Ghani AC, Lyons E, Drakeley CJ (2009). Submicroscopic infection in *Plasmodium falciparum*-endemic populations: a systematic review and meta-analysis. J Infect Dis.

[5] Okell LC, Bousema T, Griffin JT, Ouedraogo AL, Ghani AC, Drakeley CJ (2012). Factors determining the occurrence of submicroscopic malaria infections and their relevance for control. Nat Commun..

[6] Rodriguez-Barraquer I, Arinaitwe E, Jagannathan P, Kamya MR, Rosenthal PJ, Rek J (2018). Quantification of anti-parasite and anti-disease immunity to malaria as a function of age and exposure. Elife..

[7] Gosling RD, Okell L, Mosha J, Chandramohan D (2011). The role of antimalarial treatment in the elimination of malaria. Clin Microbiol Infect.

[8] WHO (2018). Meeting report of the WHO Evidence Review Group on mass drug administration for malaria: 11–13, Geneva, Switzerland.

[9] WHO (2017). Guidelines: alternative mass drug administration regimens to eliminate lymphatic filariasis.

[10] WHO (2012). Accelarating work to overcome the global impact of neglected tropical diseases: a roadmap for implementation.

[11] WHO (2006). Preventive chemotherapy in human helminthiasis: coordinated use of anthelmintic drugs in control interventions: a manual for health professionals and programme managers.

[12] Escudie A, Hamon J, Schneider J (1960). Results of mass antimalarial chemoprophylaxis with a combination of 4-aminoquinoline and 8-aminoquinoline under rural African conditions in the region of Bobo-Dioulasso (Upper Volta) 1960. Comparative study in a zone treated with DDT and outside this zone] (in French). Med Trop (Mars)..

[13] Jones SA (1958). Mass treatment with pyrimethamine; a study of resistance and cross resistance resulting from a field trial in the hyperendemic malarious area of Makueni, Kenya. September 1952–September 1953. Trans R Soc Trop Med Hyg..

[14] Molineaux L, Gramiccia G (1980). The Garki Project. Research on the epidemiology and control of malaria in the Sudan Savanna of West Africa.

[15] Singh J, Misra BG, Ray AP (1953). Suppressive treatment with amodiaquin. Indian J Malariol.

[16] Roberts JM (1956). Pyrimethamine (Daraprim) in the control of epidemic malaria. J Trop Med Hyg..

[17] Schneider J, Escudie A, Hamon J (1961). The eradication of malaria and chemotherapy. Results of a trial of the combination: “4-aminoquinoline”/”8-aminoquinoline” in the “pilot zone” of Bobo-Dioulasso (Haute-Volta) (in French). Bull Soc Pathol Exot..

[18] von Seidlein L, Greenwood BM (2003). Mass administrations of antimalarial drugs. Trends Parasitol..

[19] Poirot E, Skarbinski J, Sinclair D, Kachur SP, Slutsker L, Hwang J (2013). Mass drug administration for malaria. Cochrane Database Syst Rev..

[20] Newby G, Hwang J, Koita K, Chen I, Greenwood B, von Seidlein L (2015). Review of mass drug administration for malaria and its operational challenges. Am J Trop Med Hyg.

[21] Kaneko A, Taleo G, Kalkoa M, Yamar S, Kobayakawa T, Bjorkman A (2000). Malaria eradication on islands. Lancet.

[22] Deng C, Huang B, Wang Q, Wu W, Zheng S, Zhang H (2018). Large-scale artemisinin–piperaquine mass drug administration with or without primaquine dramatically reduces malaria in a highly endemic region of Africa. Clin Infect Dis.

[23] Eisele TP, Bennett A, Silumbe K, Finn TP, Chalwe V, Kamuliwo M (2016). Short-term impact of mass drug administration with dihydroartemisinin plus piperaquine on malaria in Southern Province Zambia: a cluster-randomized controlled trial. J Infect Dis.

[24] von Seidlein L, Peto TJ, Landier J, Nguyen TN, Tripura R, Phommasone K (2019). The impact of targeted malaria elimination with mass drug administrations on falciparum malaria in Southeast Asia: a cluster randomised trial. PLoS Med..

[25] Landier J, Kajeechiwa L, Thwin MM, Parker DM, Chaumeau V, Wiladphaingern J (2017). Safety and effectiveness of mass drug administration to accelerate elimination of artemisinin-resistant falciparum malaria: a pilot trial in four villages of Eastern Myanmar. Wellcome Open Res..

[26] Zambia Ministry of Health (2017). National Malaria Elimination Strategic Plan 2017–2021: a strategy to move from accelerated burden reduction to malaria elimination in Zambia (Brief).

[27] Parker DM, Tun STT, White LJ, Kajeechiwa L, Thwin MM, Landier J (2019). Potential herd protection against *Plasmodium falciparum* infections conferred by mass antimalarial drug administrations. Elife..

[28] Bretscher MT, Griffin JT, Ghani AC, Okell LC (2017). Modelling the benefits of long-acting or transmission-blocking drugs for reducing *Plasmodium falciparum* transmission by case management or by mass treatment. Malar J..

[29] Brady OJ, Slater HC, Pemberton-Ross P, Wenger E, Maude RJ, Ghani AC (2017). Role of mass drug administration in elimination of *Plasmodium falciparum* malaria: a consensus modelling study. Lancet Glob Health..

[30] Payne D (1988). Did medicated salt hasten the spread of chloroquine resistance in Plasmodium falciparum?. Parasitol Today..

[31] WHO (1970). Expert Committee on malaria: fifteenth report.

[32] Zuber JA, Takala-Harrison S (2018). Multidrug-resistant malaria and the impact of mass drug administration. Infect Drug Resist..

[33] Kaehler N, Adhikari B, Cheah PY, Day NPJ, Paris DH, Tanner M (2019). The promise, problems and pitfalls of mass drug administration for malaria elimination: a qualitative study with scientists and policymakers. Int Health..

[34] Chan XHS, Win YN, Mawer LJ, Tan JY, Brugada J, White NJ (2018). Risk of sudden unexplained death after use of dihydroartemisinin–piperaquine for malaria: a systematic review and Bayesian meta-analysis. Lancet Infect Dis..

[35] Morris U, Msellem MI, Mkali H, Islam A, Aydin-Schmidt B, Jovel I (2018). A cluster randomised controlled trial of two rounds of mass drug administration in Zanzibar, a malaria pre-elimination setting-high coverage and safety, but no significant impact on transmission. BMC Med..

[36] von Seidlein L, Dondorp A (2015). Fighting fire with fire: mass antimalarial drug administrations in an era of antimalarial resistance. Expert Rev Anti Infect Ther..

[37] White NJ (2017). Does antimalarial mass drug administration increase or decrease the risk of resistance?. Lancet Infect Dis..

[38] Carter R, Mendis KN, Roberts D (2000). Spatial targeting of interventions against malaria. Bull World Health Organ.

[39] Bousema T, Griffin JT, Sauerwein RW, Smith DL, Churcher TS, Takken W (2012). Hitting hotspots: spatial targeting of malaria for control and elimination. PLoS Med..

[40] Cohen JM, Le Menach A, Pothin E, Eisele TP, Gething PW, Eckhoff PA (2017). Mapping multiple components of malaria risk for improved targeting of elimination interventions. Malar J..

[41] WHO (2017). A framework for malaria elimination.

[42] WHO (2015). The role of mass drug administration, mass screening and treatment, and focal screening and treament for malaria.

[43] Adhikari B, James N, Newby G, von Seidlein L, White NJ, Day NP (2016). Community engagement and population coverage in mass anti-malarial administrations: a systematic literature review. Malar J..

[44] Hetzel MW, Genton B (2018). Mass drug administration for malaria elimination: do we understand the settings well enough?. BMC Med..

